# The Pathogenetic Picture

**Published:** 1891-07

**Authors:** B. Fincke

**Affiliations:** President of the New York Homœopathic Union, at its Third Anniversary (Founded April 19th, 1888)


### THE PATHOGENETIC PICTURE (*ORGANON*, § 83 Et Seq.).

Address of B. Fincke, M. D., President of the New York Homœopathic Union, at its Third Anniversary (Founded April 19th, 1888).

In looking upon the past year of our endeavor to study *The Organon*, it must be acknowledged that the time bestowed upon it has not been employed in vain, and those who have taken part in our discussions will testify that many subjects have been cleared up which gave to our meetings a more practical character than the year before. We had naturally to begin at the beginning, at the principles which form the basis of our science and art, and the patience and endurance with which the members went through the difficult task of understanding them clearly deserves all praise. Though some points in the explanation of the phenomena in relation to the action of medicines upon the organism could not be accepted literally as they stand, they have only led to views which already have been held by the older physicians without being sufficiently cleared up. Those doubtful points, on continued investigation, will probably be replaced by undoubted ones, in uniformity with the immutable principles laid down in the first part of *The Organon.*

We have gone through a remarkable period of commotion in the allopathic ranks, after we had settled, the year before, what there may be acceptable of so-called "Isopathy." It was to be expected that the homœopathic ranks would be carried away with it more or less. Our homœopathic brethren on the other side of the Atlantic, in consequence, raised the formerly by them despised corpse of Isopathy from its death and galvanized it into new life, because by these means they could declare Koch's method a homœopathic measure. In a paper read before the Union at the last December meeting, it was shown what a sorry homœopathic measure that was and its downfall was predicted, because it lacked the true scientific element of induction, which invariably demands the proving of the medicine upon the healthy and the proper potentiation of it to make it acceptable to the life-force when sick, before it can be administered in disease. Naturally, no allusion was made to this extravaganza of the physico-chemical school in our meetings, when it was at its height, because we had settled the matter before in the claim that such morbid substances (nosodes), if preferred, should be obtained in their purity of corruption. They should be potentiated and proved upon the healthy, and then they should be considered to be homœopathic remedies like the rest, inasmuch as then they cease to be nosodes.

One would think that the fiasco of Koch's lymph would have opened the people's eyes at large, so that they would see how the inveterate enemies of Homœopathy blundered in adopting it in an allopathic way, and thus recognize the poverty of testimony given thereby. But it is idle to suppose that the majority of the people would take a lesson from that humiliating occurrence in the allopathic camp. It is even so, that in respect to medical matters great darkness prevails in the minds of even intelligent men and women, and no homœopathic ray of salvation can enter there. We are still a small minority, and if it were not that the light, which Hahnemann has wrought out of solid facts, shines of its own accord even in the darkness around, we might despair that it ever would overcome it! But there is a great comfort in the struggle in which we are engaged. Homœopathy, at the same time that it carries the light through the darkness, comforts and heals the sick and spreads its blessings around in its march toward greater enlightenment of the human race.

We have now in *The Organon* arrived at the sections which teach how to investigate the given case of disease. Never was there anything written more to the point, though extensive enough to cover all possible conditions which the sick to be examined can present. Hahnemann in these sections proves himself to be as great a pedagogue as he was a physician, and enables every sensible physician to understand the case before him—*i. e.*, what of it he needs to know for healing it, even if he has not the mind of a Hahnemann. If the Hahnemannians are called sectarians and their teaching exclusive, this is only in vulgar parlance, "calling names." In doing so, they in reality do not mean us personally, but the master in whose tracks we are proud to follow. They try to blind their contemporaries with representing Hahnemann before he arrived at the perfection of his doctrine as it is laid down in the fifth edition of *The Organon*, thirty-three years after the first edition. All the great reformers had the same fate of being reviled at first; and how could it be otherwise? They had to rise above the common level of the human understanding, because they discovered phenomena and facts which had not yet or only imperfectly been observed. When Copernicus set the sun in the middle of our world as *laterna mundi*, around which the planets move in circles drawn by a mysterious attraction; when Kepler rectified the circular by the elliptical motion of the planets, and Newton crowned the work of these two great men by his discovery of the law of universal gravitation, they had to encounter the enmity of their contemporaries, who were in the great majority and railed at the presumption of single obscure individuals who dared to overthrow the error of ages held by celebrated men.

For, before the arrival at their new discoveries, they were unknown to the fame which praised them afterward to the skies. Just so Hahnemann. What matters it if they call him sectarian and exclusive, and his faithful disciples an exclusive sect? His discoveries go through all ages to come, gaining continually by their industrious labors in extent and intensity. For the homœopathic school is different from the old school in this, that may the various sciences be perfected in the course of time ever so much, the science of homœopathics proceeds on its own way, pursuing the perfection of its own methods and increasing in usefulness in the healing of the sick, however much the hypotheses and theories cultivated by the physico-chemical school may vary. If the various auxiliary sciences of medicine proceed incessantly accumulating facts and knowledge, it does not follow that their teaching is also progress. Frequently they are the cause of new methods which are introduced as improvements, and afterward turn out to be the reverse. Look at the methods of blood-letting, of stimulation, of vaccination, of injecting morbid products as we have seen lately. All these perilous efforts pass like storm-winds, leaving desolation behind them. But Homœopathy, based upon sound principles, continues its even way, rendering to the diligent mind the means to heal where healing is possible.

Such thoughts keep coming up when we consider the careful prescription which Hahnemann gives for the exact investigation of the case in hand. And it stands to reason that, if the homœopathician has acquired the faculty and skill to take up the pathogenetic picture in the Hahnemannian manner, he will be just as careful in selecting the similar remedy for it which the *Materia Medica Pura* offers.

Let us, therefore, my friends, not get tired at the numerous details which some cases present. Their evolution is the best assurance of a successful selection of the similar remedy, and thus the conscientious study of *The Organon* will be not only a great benefit for our practice, but also a pleasure in exercising and perfecting the mind.

